# Circulating B cells display differential immune regulatory molecule expression in granulomatosis with polyangiitis

**DOI:** 10.1093/cei/uxae096

**Published:** 2024-10-21

**Authors:** Carlo G Bonasia, Nanthicha Inrueangsri, Theo Bijma, Malte Borggrewe, Aline I Post, Kevin P Mennega, Wayel H Abdulahad, Abraham Rutgers, Nicolaas A Bos, Peter Heeringa

**Affiliations:** Department of Rheumatology and Clinical Immunology, University Medical Center Groningen, University of Groningen, Groningen, The Netherlands; Department of Pathology and Medical Biology, University Medical Center Groningen, University of Groningen, Groningen, The Netherlands; Department of Pathology and Medical Biology, University Medical Center Groningen, University of Groningen, Groningen, The Netherlands; Independent Data Lab, London, UK; Department of Pathology and Medical Biology, University Medical Center Groningen, University of Groningen, Groningen, The Netherlands; Department of Pathology and Medical Biology, University Medical Center Groningen, University of Groningen, Groningen, The Netherlands; Department of Rheumatology and Clinical Immunology, University Medical Center Groningen, University of Groningen, Groningen, The Netherlands; Department of Pathology and Medical Biology, University Medical Center Groningen, University of Groningen, Groningen, The Netherlands; Department of Rheumatology and Clinical Immunology, University Medical Center Groningen, University of Groningen, Groningen, The Netherlands; Department of Rheumatology and Clinical Immunology, University Medical Center Groningen, University of Groningen, Groningen, The Netherlands; Department of Pathology and Medical Biology, University Medical Center Groningen, University of Groningen, Groningen, The Netherlands

**Keywords:** vasculitis, B cells, ANCA, spectral flow cytometry

## Abstract

Granulomatosis with polyangiitis (GPA) is a B-cell-mediated, relapsing, autoimmune disease. There is a need for novel therapeutic approaches and relapse markers to achieve durable remission. B cells express immune regulatory molecules that modulate their activation and maintain tolerance. While recent studies show dysregulation of these molecules in other autoimmune diseases, data on their expression in GPA are limited. This study aimed to map the expression of surface immune regulatory molecules on circulating B-cell subsets in GPA and correlate their expression with clinical parameters. Immune regulatory molecule expression on circulating B-cell subsets was comprehensively examined in active GPA (*n* = 16), GPA in remission (*n* = 16), and healthy controls (*n* = 16) cross-sectionally using a 35-color B-cell-specific spectral flow cytometry panel.

Our supervised and unsupervised in-depth analysis revealed differential expression of inhibitory and stimulatory immune molecules on distinct B-cell populations in GPA, with the most notable differences observed in active GPA. These differences include the upregulation of FcγRIIB on nonmature B cells, downregulation of CD21 and upregulation of CD86 on antigen-experienced B cells, and elevated CD22 expression on various populations. Additionally, we found a strong association between FcγRIIB, BTLA, and CD21 expression on specific B-cell populations and disease activity in GPA.

Together, these findings provide novel insights into the immune regulatory molecule expression profile of B cells in GPA and could potentially form the foundation for new therapeutic approaches and disease monitoring markers.

## Teaser

Spectral flow cytometry uncovers a distinct B-cell composition profile in granulomatosis with polyangiitis (GPA). GPA patients have altered expression of FcγRIIB, CD21, CD22, and CD86 on B cells. FcγRIIB, BTLA, and CD21 expression on B cells are strongly associated with disease activity in GPA.

## Graphical Abstract

**Graphical Abstract:**
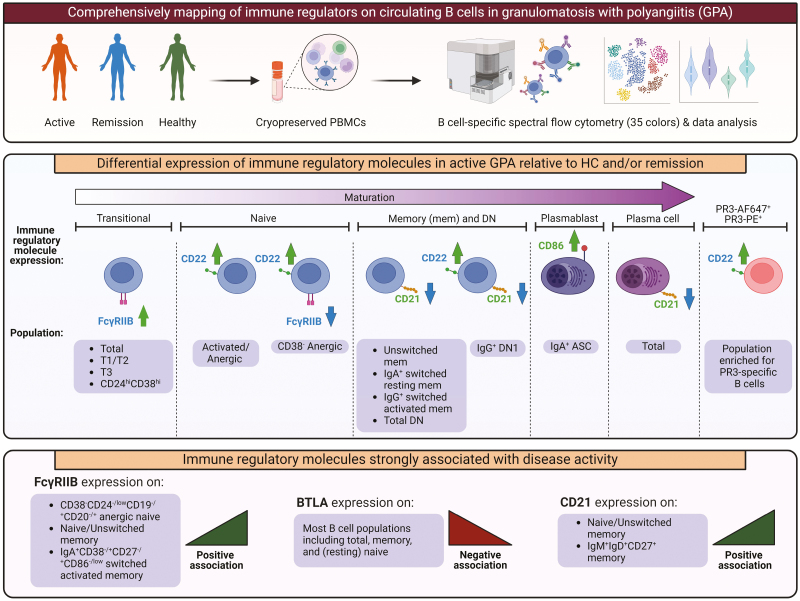

## Introduction

Granulomatosis with polyangiitis (GPA) is a B-cell-mediated, systemic, autoimmune disease characterized by necrotizing granulomatous inflammation of small blood vessels frequently affecting the airways and kidneys [1]. GPA, along with microscopic polyangiitis (MPA) and eosinophilic GPA, comprises the antineutrophil cytoplasmic antibody (ANCA)-associated vasculitides (AAV). The autoimmune response in GPA targets proteinase 3 (PR3), a protease stored in neutrophil granules and monocyte lysosomes [2]. Importantly, the clinical course of GPA is defined by periods of unpredictable relapses that may result in accumulating irreversible organ damage and increased mortality [3]. Although corticosteroids, immunosuppressants, and biologics often induce clinical remission, relapses are common upon treatment cessation, and long-term therapy poses risks of infection and reduces vaccine efficacy. Moreover, reliable biomarkers for predicting relapses in GPA remain elusive. Factors such as nasal *Staphylococcus aureus* carriage [4, 5], rising or high PR3-ANCA levels [6–8], and lower serum creatinine levels at the time of diagnosis [9] have been linked to higher relapse risks. Additionally, specific cellular biomarkers like low CD5^+^ B-cell frequencies following rituximab therapy [10] and high CD27^+^CD38^hi^ B-cell frequencies during remission [11] have been associated with relapses, albeit with limited clinical applicability. Therefore, novel therapeutic approaches and relapse risk markers need to be identified to achieve and maintain durable remission in GPA patients.

B cells play a crucial role in the pathogenesis of GPA. This is evident from the loss of B-cell tolerance to PR3 as demonstrated by the presence of circulating PR3-ANCAs in GPA and the occurrence of a higher frequency of circulating PR3-specific B cells in GPA patients compared to healthy controls (HCs) [1, 12–14]. In line, PR3-ANCAs are considered to be pathogenic in GPA by activating neutrophils leading to the release of inflammatory mediators by neutrophils causing vascular damage [1]. Besides PR3-ANCA production, B cells may also contribute to GPA pathogenesis through antibody-independent effector functions such as cytokine production and antigen presentation [15, 16]. Importantly, the specific depletion of B cells by rituximab, a chimeric monoclonal antibody targeting CD20, is highly effective in inducing clinical remission [17–19].

A diverse array of immune stimulatory and inhibitory molecules are expressed by B cells which collectively modulate their activation, tolerance, and effector functions [20]. These immune regulatory molecules include CD40, toll-like receptors, cytokine receptors, programmed cell death 1 (PD-1), CD22, and others [20]. Maintaining a balance between positive and negative influences on B-cell function, through these immune regulatory molecules, is critical at all stages of B-cell development as an imbalance can contribute to autoimmunity and autoinflammatory processes [20, 21].

Intriguingly, the expression of immune regulatory molecules on B cells appears to be dysregulated in various autoimmune diseases. Lower expression of the inhibitory receptor Fc fragment of IgG receptor IIb (FcγRIIB) has been observed on circulating CD27^+^ B cells [22] and circulating marginal zone B cells [23] in systemic lupus erythematosus (SLE). In addition, the expression of the inhibitory receptor B- and T-lymphocyte attenuator (BTLA) has been reported to be reduced on circulating naive B cells in SLE [24]. Moreover, circulating autoreactive memory B cells in rheumatoid arthritis (RA) showed a higher expression of the stimulatory receptor CD40 compared to nonautoreactive B cells [25]. Data on the expression of immune regulatory molecules in AAV are scarce. A recent study reported reduced expression of FcγRIIB on circulating plasma cells in an AAV cohort mostly consisting of MPA patients, which was associated with disease activity [26]. In general, dysregulation of immune regulatory molecules on B cells is associated with a reduction of the B-cell activation threshold, inappropriate B-cell expansion and survival, B-cell tolerance escape, and aberrant B-cell effector functions [22–24]. Hence, identifying changes in the expression of immune-modulatory molecules on B cells in GPA is important, as they could help uncover novel therapeutic targets or lead to predictive biomarkers for disease progression.

In this study, we comprehensively mapped the expression of surface immune regulators on circulating B cells in patients with active GPA and those in clinical remission, leveraging a 35-color B-cell-specific spectral flow cytometry panel, and compared it with HCs. We also explored the association between the expression profiles of these immune regulators and clinical indicators, including Birmingham Vasculitis Activity Score (BVAS) and PR3-ANCA levels. Through detailed analysis of these associations, we aimed to uncover insights that could lay the foundation for the discovery of new therapeutic approaches and disease monitoring markers.

## Materials and methods

### Study population

A comprehensive overview of the patient and HC characteristics is provided in Table 1. Peripheral blood samples were cross-sectionally collected from GPA patients with active disease (*n* = 16), GPA patients in clinical disease remission (*n* = 16), and age- and sex-matched HCs (*n* = 16) using lithium heparin-coated vacutainers (BD Biosciences, San Jose, CA). Of these, four patients were sampled in both active and remission disease states. Seven patients in the active disease group were at disease onset. The study received approval from the Institutional Review Board of the University Medical Center Groningen (METc no. 2012/151 and 2020/007), and written informed consent was obtained from all donors. The diagnosis of GPA in patients followed the Chapel Hill Consensus Conference definitions and met the classification criteria of the American College of Rheumatology [27, 28]. Patients who were undergoing immunosuppressive treatment at the time of sampling were excluded from the study. Eligibility for inclusion required a B-cell frequency of >1% within the single live lymphocyte populations of peripheral blood mononuclear cells (PBMCs).

**Table 1:** patient and healthy control characteristics

	Active	Remission	HC
Total, *n*	16	16	16
Sex (% male)	50.0	50.0	50.0
Age, mean (range)	63.0 (30–77)	63.2 (44–75)	63.0 (47–80)
PR3-ANCA (U/ml), median (range)	46.0 (4.1–177)	16.5 (0.0–177)	
CRP (mg/l), median (range)	41.0 (0.4–172)	3.4 (0.4–16)	
Creatinine (μmol/l), median (range)	83.5 (62.0–483)	83.0 (58–178)	
eGFR (ml/min/1,73m^2^), median (range)	70 (11–98)	72 (32–103)	
Disease onset, *n* (%)	7 (44)	-	
Disease duration (years), median (range)	4.9 (0.0–18.2)	9.8 (3.0–21.0)	
BVAS, median (range)	14 (6–20)	0 (-)	
Organ involvement, *n* (%)			
Kidney	7 (44)	7 (44)	
Ear/nose/throat	11 (69)	12 (75)	
Joint	6 (38)	7 (44)	
Lung	10 (63)	7 (44)	
Eye	3 (19)	6 (38)	
Nervous system	4 (25)	4 (25)	
Skin	2 (13)	2 (13)	
Other	0 (0)	0 (0)	
Disease form, *n* (%)			
Localized	2 (13)	2 (13)	
Early systemic	3 (19)	3 (19)	
Generalized	11 (69)	11 (69)	
Severe	0 (0)	0 (0)	
Medication at sampling time point, *n* (%)	0 (0)	0 (0)	
Prior medication^a^, *n* (%)			
Prednisone	3 (19)	4 (25)	
Cyclophosphamide	0 (0)	0 (0)	
Rituximab^b^	3 (19)	0 (0)	
Azathioprine	0 (0)	3 (19)	
Methotrexate	0 (0)	1 (6)	

^a^Last immunosuppressive medication up to 3 years before blood sampling time point.

^b^Three patients in the active GPA group received rituximab within 3 years prior to blood sampling, with treatment intervals of 1 year and 2 months, 1 year and 3 months, and 1 year and 4 months. Additionally, a fourth active patient received rituximab 8 years and 8 months before sampling.

Abbreviations: BVAS: Birmingham Vasculitis Activity Score; CRP: C-reactive protein; eGFR: estimated glomerular filtration rate; HC: healthy control; PR3-ANCA: proteinase 3-anti-neutrophil cytoplasmic antibodies.

### Peripheral blood mononuclear cell isolation

PBMCs were obtained from lithium heparin-treated blood through density gradient centrifugation using Lymphoprep (Stemcell, Vancouver, Canada). The isolated PBMCs were cryopreserved in Roswell Park Memorial Institute 1640 Medium (RPMI 1640, Lonza, Basel, Switzerland), which was supplemented with 10% FBS, 10% dimethyl sulfoxide, and 50 μg/ml gentamycin (Lonza) and then followed by controlled overnight freezing to –80°C before storage in liquid nitrogen.

### Spectral flow cytometry panel

A 35-color spectral flow cytometry panel was developed to enable the in-depth phenotyping of circulating B cells, along with the mapping of surface immune regulatory molecule expression. A detailed overview of the panel is listed in Supplementary Table 1. The panel was built by expanding the published 24-color B-cell-specific optimized multicolor immunofluorescence panel-68 (OMIP-68) [29] with additional markers for B-cell lineage (IgG, IgA, and CD138) and stimulatory molecules (CD40, inducible costimulator ligand [ICOSL], B-cell activating factor receptor [BAFFR], transmembrane activator and CAML interactor [TACI], IL-21R, and IL-6R). Native human PR3, which was originally isolated from the granules of leukocytes obtained from buffy coats of healthy individuals [2], labeled with AlexaFluor647 (AF647) or R-phycoerythrin (PE), was also included in the panel for the detection of a B-cell population enriched for PR3-specific B cells. A dual-staining strategy using PR3-AF647 and PR3-PE was previously tested and optimized on hybridoma cells (Supplementary Fig. 1). Prior to staining, antibody titrations, testing, and optimization were conducted on cryopreserved PBMCs from HCs and GPA patients.

### Staining of PBMCs for spectral flow cytometry

Staining of PBMCs was performed using an adapted version of the method described in OMIP-68 [29]. Briefly, PBMCs were thawed in RPMI 1640 supplemented with 10% FCS and 50 μg/ml gentamycin followed by a washing step with PBS. Per sample, 2 × 10^6^ PBMCs were incubated with LIVE/DEAD Fixable Blue Stain (Thermo Fisher Scientific, Waltham, MA) for 15 minutes at room temperature (RT). Cells were washed with PBS and afterward incubated with Brilliant Stain Buffer Plus (BD Biosciences) and True-Stain Monocyte Blocker (BioLegend, San Diego, CA). The staining process involved a prestaining step with anti-IL-6R-BV421, anti-ICOSL-BB790-P, anti-CD11c-BV650, and anti-TACI-PE-Dazzle 594 of 15 min. at RT. Subsequently, cells were incubated with the remaining antibodies (Supplementary Table 1) for 30 minutes at RT, followed by washing with PBS/1% BSA and fixation with FACS Lysing Solution (BD Biosciences) for 20 minutes at RT. After centrifugation and resuspension in PBS/1% BSA, 1 × 10^6^ PBMCs were acquired using a 5-laser Cytek Aurora full spectrum flow cytometer (Cytek Biosciences, Fremont, CA). Samples from different groups were randomly distributed across batches to mitigate technical variation among the groups.

### Spectral flow cytometry data analysis through manual gating

**Figure 1:**
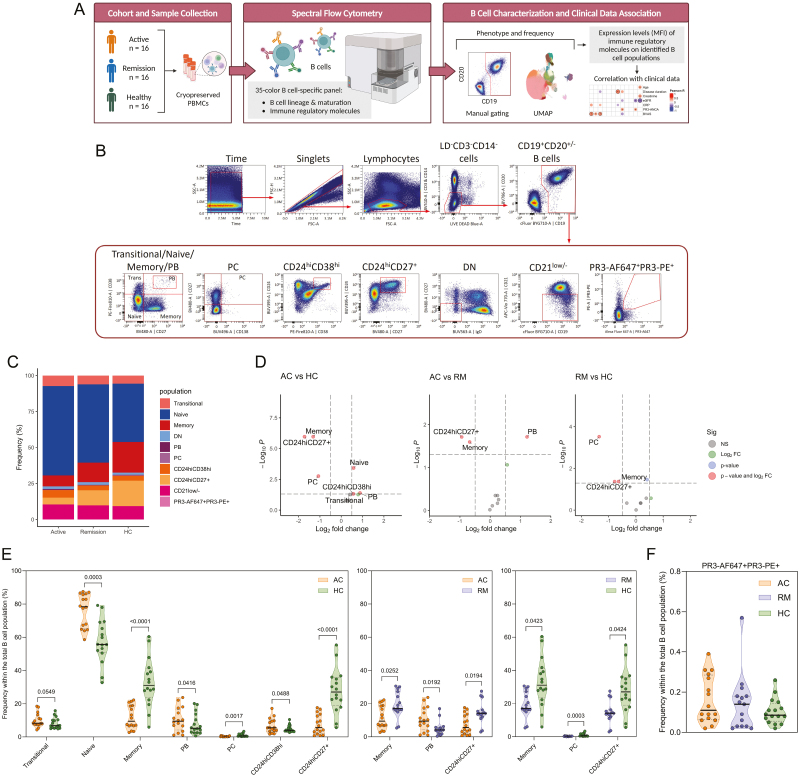
distribution of major B-cell subsets is aberrant in GPA. (a) Schematic overview of the experimental approach. (b) Gating strategy for the identification of the major B-cell subsets. The figure depicts a representative staining of PBMCs from one GPA patient in remission. (c) Frequency plot showing the distribution of major B-cell subsets within the total B-cell population of each group (active, *n* = 16; remission, *n* = 15; HC, *n* = 16). (d) Volcano plots displaying the results of the differential abundance analysis. Cell populations demonstrating a *P*adj-value <0.05 and a log fold change >0.5 are highlighted. (e) Violin plots depicting the differences in the frequencies of major B-cell subsets among the groups identified through manual gating analysis. Only plots depicting significant differences in B-cell population frequencies between the groups are shown. The median is shown by a black line, and the interquartile range (IQR) is shown by dotted lines. (f) Violin plots depicting the frequency of PR3-AF647^+^PR3-PE^+^ B cells among the groups. Likelihood ratio test, BH correction. AC: active; DN: double negative; PB: plasmablasts; PC: plasma cells; HC: healthy control; RM: remission.

Spectral flow cytometry data were analyzed using manual gating in OMIQ Flow Cytometry Software (www.omiq.ai, Dotmatics) complemented by statistical analysis in GraphPad Prism version 10 (GraphPad Software, La Jolla, CA). CD19^+^CD20^+/-^ B cells were gated from single live CD3^-^CD14^-^ cells (Fig. 1b). Classification of major B-cell subsets was based on the current B-cell classification literature, with CD19^+^CD20^+/−^ B cells categorized in transitional (CD27^−^CD38^hi^), naive (CD27^−^CD38^−/low^), memory (CD27^+^CD38^−/low^), plasmablasts (CD27^hi^CD38^hi^), and plasma cells (CD27^+^CD138^+^) [11]. In addition, CD19^+^CD20^+/−^ B cells were classified into double-negative (CD27^−^IgD^−^), CD21^low/−^ B cells, two putative regulatory B-cell subsets (CD24^hi^CD38^hi^, enriched within the transitional B-cell population, and CD24^hi^CD27^+^, enriched within the memory B-cell population) [30], and a population enriched for PR3-specific B cells (PR3-AF647^+^PR3-PE^+^).

### High-dimensional data reduction

Spectral flow cytometry data were also analyzed through high-dimensionality reduction and visualization in OMIQ Flow Cytometry Software and GraphPad Prism version 10. Single live CD3^−^CD14^−^CD19^+^CD20^+/−^ B cells were manually gated from each sample with subsequent data concatenation amounting to 1.594.511 cells in total. Batch normalization was performed using fdaNorm. Uniform Manifold Approximation and Projection (UMAP) and FlowSOM algorithms were applied for data dimensionality reduction, visualization, and clustering. Parameters for UMAP and FlowSOM are detailed in Supplementary Table 2. Annotation of B-cell populations was based on established B-cell classification literature [31].

### Differential abundance analysis

The edgeR package [32] was employed for the differential abundance analysis of cell populations identified through manual gating and UMAP. This process included a normalization step to adjust for total cell counts per donor, followed by the creation of a generalized linear model that incorporated cell numbers for each population/cluster and donor across different groups. One patient in remission was excluded from this analysis and subsequent analyses due to aberrant frequencies of various B-cell populations, resulting in outliers (e.g. 79.2% CD21^−/low^ B cells within the total B-cell population versus a median frequency of 13.9% (range: 8.0–22.9%) in the remission group (excluding the outlier), 14.4% (range: 8.0–22.9%) in the active group, and 12.7% (range: 5.6–27.6%) in the HC group.

### Differential expression analysis

Differential expression analysis was performed using the limma R package [33]. Analysis was conducted using log-transformed median fluorescence intensity (MFI) values derived from manually gated populations and UMAP. IL-21R and IL-6R were excluded from the analysis due to their weak signals, resulting in unreliable results. MFI values for each immune regulatory molecule were analyzed across all B cells for broadly expressed molecules (FcγRIIB, CD22, BTLA, CD40, BAFFR, and CD21) and within molecule-positive cells for molecules expressed by a fraction of B cells (PD-1, FcRL5, CD5, ICOSL, TACI, and CD86). Populations containing fewer than 10 cells, or datasets where more than 20% of the data from a group was missing due to low cell counts, were excluded from analysis.

### Statistical analysis

Statistical analyses were conducted using the programming language R with RStudio and GraphPad Prism version 10. For the differential abundance analysis, pairwise comparisons were conducted using a likelihood ratio test to identify significant differences in abundance between groups. To mitigate the risk of type I errors due to multiple testing, *P*-values were adjusted using the Benjamini–Hochberg (BH) method, and the results were reported as false discovery rates. The differential expression analysis was performed using the limma R package [33], where a linear model was fitted and empirical Bayes statistics (eBayes) were utilized to determine differential expression. Correlations between the expression of immune regulators on B-cell populations and clinical parameters were assessed using the Pearson correlation coefficient. The strength of the correlation was categorized as weak (<0.4), moderate (0.4–<0.6), strong (0.6–0.8), or excellent (>0.8). Adjustments for multiple comparisons were also made using the BH correction. Data visualization through heatmaps employed Ward’s agglomerative hierarchical clustering. *P*-values 0.05 were considered statistically significant for all tests.

## Results

### Circulating B-cell compartment in GPA varies primarily in naive and memory populations

To delineate the composition of the circulating B-cell compartment among GPA patients with active disease, those in remission, and HCs, PBMC samples were immunophenotyped using the 35-color B-cell-specific spectral flow cytometry panel, and frequencies were determined (Fig. 1a and Supplementary Table 1).

Differential abundance analysis of the major B-cell subsets, identified through manual gating (Fig. 1b and Supplementary Fig. 2), revealed an elevated frequency of naive B cells, plasmablasts, and CD24^hi^CD38^hi^ B cells and a tendency toward increased transitional B cells within the total B-cell population in active GPA compared to HC (Fig. 1c–e and Supplementary Fig. 3). Conversely, the frequency of memory B cells, plasma cells, and CD24^hi^CD27^+^ B cells was reduced in active GPA. Between active GPA and remission, the frequency of plasmablasts was higher in active, while the frequency of memory B cells and CD24^hi^CD27^+^ B cells was lower. Patients in remission were characterized by a lower frequency of memory B cells, plasma cells, and CD24^hi^CD27^+^ B cells compared to HCs. Analysis of PR3-AF647^+^PR3-PE^+^ B cells within the total B-cell population showed a higher frequency in a subset of GPA patients than HCs regardless of the disease state, although this difference did not reach statistical significance (Fig. 1f).

**Figure 2:**
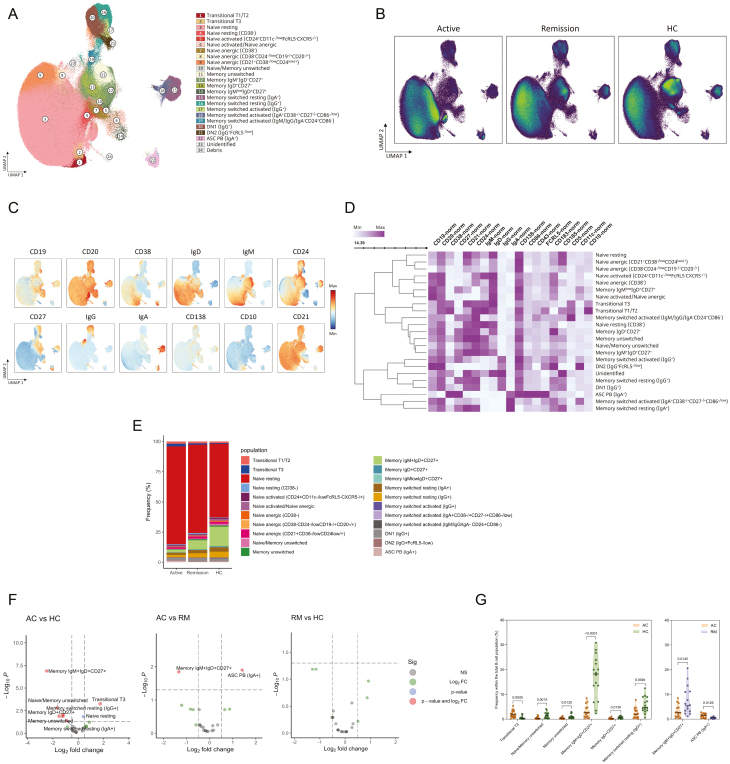
circulating B-cell compartment in GPA varies primarily in naive and memory populations. (a) UMAP of B cells with FlowSOM overlay, generated using 1.594.511 B cells derived from merged samples of GPA patients with active disease (*n* = 16), remission (*n* = 15), and HC (*n* = 16). (b) UMAP density plot for each group. (c) UMAP plots showing the normalized expression of major B-cell markers. (d) Heatmap visualizing marker expression profiles across B-cell populations with min–max normalization for scaling, created using Ward’s agglomerative hierarchical clustering. (e) Frequency plot showing the distribution of B-cell populations within the total B-cell population of each. (f) Volcano plots displaying the results of the differential abundance analysis. Cell populations demonstrating a *P*adj-value <0.05 and a log fold change >0.5 are highlighted. (g) Violin plots depicting the differences in the frequencies of B-cell populations among the groups identified through UMAP analysis. Only plots depicting significant differences in B-cell population frequencies between the groups are shown. The median is shown by a black line, and the IQR by dotted lines. Likelihood ratio test, BH correction. AC: active; ASC: antibody-secreting cells; DN: double negative; HC: healthy control; PB: plasmablasts; RM: remission.

We further analyzed the circulating B-cell compartment from GPA patients and HCs in-depth using a high-resolution UMAP of the spectral flow cytometry data. Data from all samples were pooled, resulting in a total of 1.594.511 B cells. A UMAP was generated using normalized markers (Supplementary Table 2), and the B-cell populations were classified according to the established classification literature [31]. With this approach, we identified 23 distinct B-cell populations (Fig. 2a–d and Supplementary Fig. 4). When comparing active GPA to HC, an increased frequency of transitional T3 B cells and a tendency toward increased resting naive B cells was observed in active GPA (Fig. 2e–g and Supplementary Fig. 5). In contrast, the frequency of naive/unswitched memory, unswitched memory, IgM^+^IgD^+^CD27^+^ memory, IgD^+^CD27^+^ memory, IgG^+^ switched resting memory, and IgA^+^ switched resting memory B cells was lower in active GPA. Active GPA displayed a higher frequency of IgA^+^ plasmablasts and a lower frequency of IgM^+^IgD^+^CD27^+^ memory B cells compared to remission. In the comparison between remission and HC, no (significant) differences were identified.

Taken together, these results demonstrate shifts within the circulating B-cell compartment in GPA, notably characterized by variability in naive and memory B-cell populations between GPA, independent of disease state, and HC.

### Immune regulatory molecules are differently expressed on B cells in GPA

Next, the expression profiles of both inhibitory (FcγRIIB, CD22, BTLA, PD-1, FcRL5, and CD5) and stimulatory (CD40, ICOSL, BAFFR, TACI, CD21, and CD86) surface immune molecules were mapped across the B-cell populations in GPA patients and HCs.

**Figure 3:**
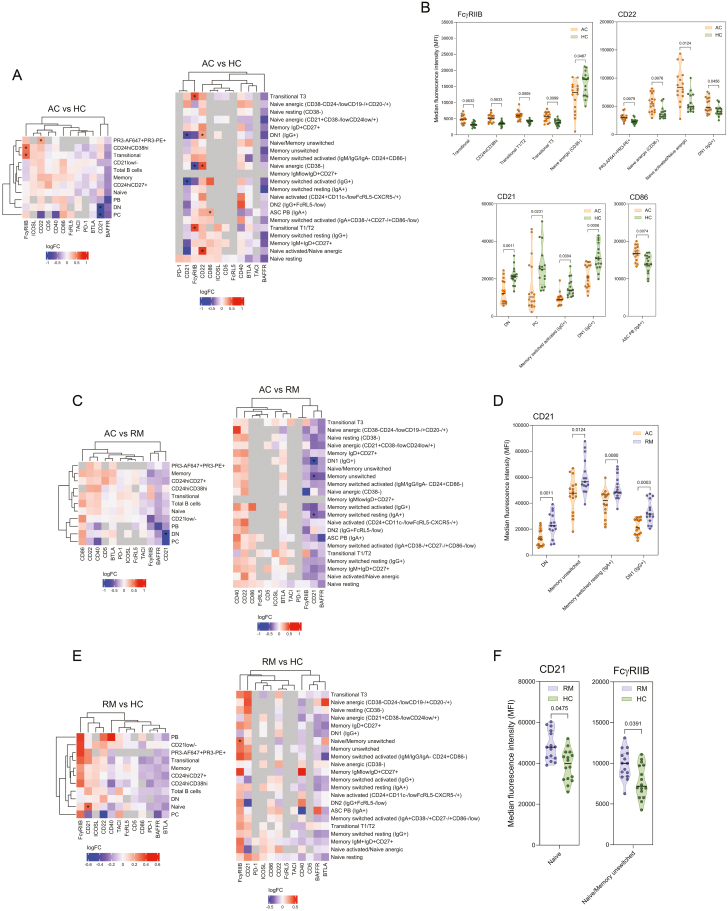
immune regulatory molecules are differently expressed on B cells in GPA. (a, c, and e) Heatmaps illustrating log2 fold changes in median fluorescence intensity (MFI) of immune regulatory molecules expressed on circulating B-cell populations across (a) active GPA (*n* = 16) and HC (*n* = 16), (C) active GPA (*n* = 16) and GPA in remission (*n* = 15), and (E) GPA in remission (*n* = 15) and HC (*n* = 16). The left heatmap depicts log2 fold changes obtained from major B-cell populations identified through manual gating, while the right heatmap displays log2 fold changes from B-cell populations obtained through UMAP analysis. Comparisons with more than 20% missing data due to low cell counts (<10 cells) were excluded from analysis and are represented in gray. (b, d, and f) Violin plots depicting the significantly different expression of regulatory molecules between (b) active and HC, (d) active GPA and GPA in remission, and (f) GPA in remission and HC. The median is shown by a black line, and the IQR by dotted lines. Asterisks indicate significant differences (*P*adj < 0.05), eBayes, BH correction. AC: active; ASC: antibody-secreting cells; DN: double negative; HC: healthy control; PB: plasmablasts; PC: plasma cells; RM: remission.

Comparative analysis of the inhibitory immune molecules between active GPA and HC demonstrated an upregulated expression of FcγRIIB in active GPA on total transitional, CD24^hi^CD38^hi^, transitional T1/T2, and transitional T3 cells, while the expression on CD38^-^ anergic naive B cells was downregulated (Fig. 3a and b). Elevated expression of CD22 in active GPA was observed on PR3-AF647^+^PR3-PE^+^, CD38^-^ anergic naive, activated naive/anergic naive, and IgG^+^ DN1 B cells. Examination of the differential expression of stimulatory immune molecules between active GPA and HC showed a lower expression of CD21 on total DN, plasma cells, IgG^+^ switched activated memory, and IgG^+^ DN1 B cells (Fig. 3a and b). CD86 expression was increased on IgA^+^ plasmablasts in active GPA. Moreover, BAFFR expression was lower across all B-cell populations in active GPA, although this difference did not reach significance (Fig. 3a).

Between active GPA and remission, the only differentially expressed molecule was CD21, which was lower expressed in several B-cell populations, including total DN, unswitched memory, IgA^+^ switched resting memory, and IgG^+^ DN1 B cells (Fig. 3c and d). Furthermore, an increased expression of CD21 on naive and FcγRIIB on naive/unswitched memory B cells was found in GPA in remission when compared with HC (Fig. 3e and f).

Overall, these observations show a differential expression of inhibitory and stimulatory immune molecules across B-cell populations in GPA, particularly in active disease.

### FcγRIIB, BTLA, and CD21 expression on B cells in active GPA strongly correlate with disease activity

Lastly, the expression of immune regulatory molecules on B cells in active GPA was correlated with clinical parameters to assess their potential relevance to disease activity.

**Figure 4:**
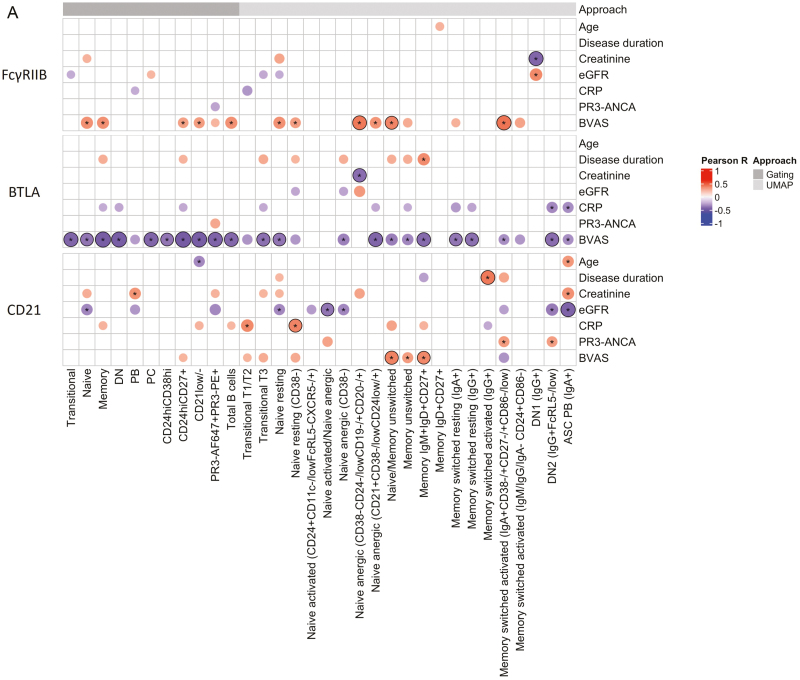

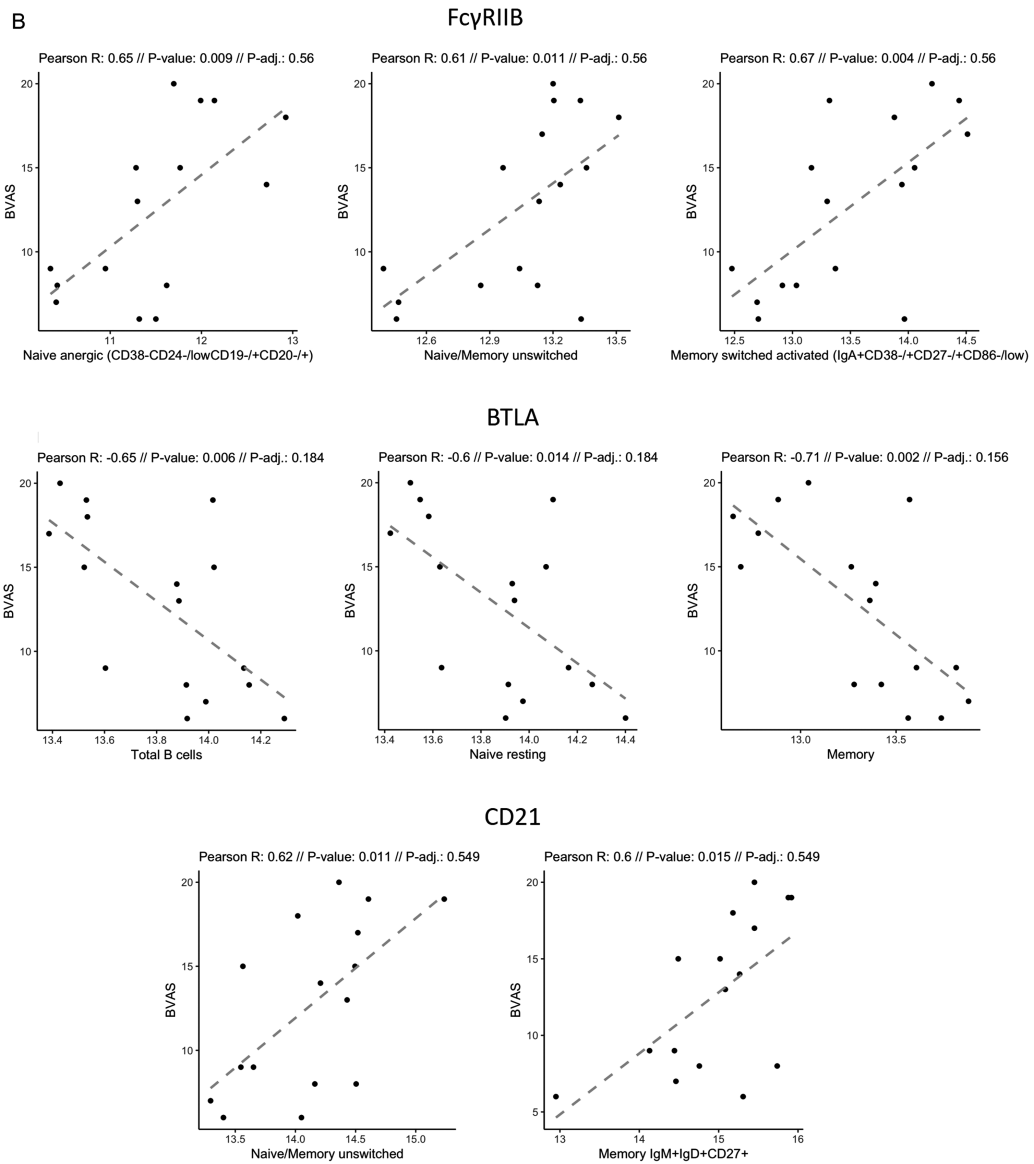
FcγRIIB, BTLA, and CD21 expression on B cells in active GPA strongly correlate with disease activity. (a) Correlation heatmap displaying the correlation of FcγRIIB, BTLA, and CD21 expression on B-cell populations with clinical parameters in active GPA (*n* = 16). The size of the dots is proportional to the correlation coefficient. Only correlations with coefficients ≥0.4 and ≤0.4 are shown, and those with coefficients ≥ 0.6 are highlighted with a black border. Asterisks indicate unadjusted *P* < 0.05. For all *P*(adj)-values, see Supplementary Data File 2. (b) Scatter plots depicting the correlation of FcγRIIB, BTLA, and CD21 expression with BVAS. For the other scatter plots showing a strong correlation between BTLA expression and BVAS, see Supplementary Fig. 6. BVAS: Birmingham Vasculitis Activity Score; CRP: C-reactive protein; eGFR: estimated glomerular filtration rate; PR3-ANCA (indicates circulating PR3-ANCA levels in this context): proteinase 3-anti-neutrophil cytoplasmic antibodies.

Correlation analysis unveiled a strong positive correlation of FcγRIIB expression on CD38^-^CD24^-/low^CD19^−/+^CD20^−/+^ anergic naive (*r* = 0.65), naive/unswitched memory (*r* = 0.61), and IgA^+^CD38^-/+^CD27^−/+^CD86^−/low^ switched activated memory (*r* = 0.67) B-cell populations with the BVAS (Fig. 4a and b; All *P*(adj)-values are provided in Supplementary Data File 2). Additionally, FcγRIIB expression on IgG^+^ DN1 B cells showed a strong negative correlation with serum creatinine levels (*r* = −0.66). BTLA expression demonstrated a strong negative correlation across a wide range of B-cell populations with the BVAS, including total (*r* = −0.65), memory (*r* = −0.71), and resting naive (*r* = −0.60) B cells (Fig. 4a and b and Supplementary Fig. 6). BTLA expression on CD38^-^CD24^-/low^CD19^−/+^CD20^−/+^ anergic naive B cells also showed a strong negative association with serum creatinine levels (*r* = −0.63). Moreover, the expression of CD21 on naive/unswitched memory (*r* = 0.62) and IgM^+^IgD^+^CD27^+^ memory (*r* = 0.60) B cells strongly correlated positively with the BVAS. CD21 expression exhibited a strong positive correlation on CD38^−^ resting naive B cells with C-reactive protein (CRP) levels (*r* = 0.61), and IgG^+^ switched activated memory B cells with disease duration (*r* = 0.65). In addition, a strong negative correlation was observed between CD21 expression on activated naive/anergic naive B cells (*r* = −0.60) and IgA^+^ PB (*r* = −0.67) and estimated glomerular filtration rate (eGFR). Furthermore, the expression of other immune regulatory molecules demonstrated no moderate correlation with the BVAS, but some exhibited a strong correlation with other clinical parameters (Supplementary Fig. 7). For instance, ICOSL expression on IgM^+^IgD^+^CD27^+^ memory B cells and IgG^+^ switched resting memory B cells showed a strong positive correlation with disease duration.

Together, these results demonstrate significant associations of FcγRIIB, BTLA, and CD21 expression on B cells with disease activity in GPA.

## Discussion

In this study, we comprehensively mapped the expression of immune regulatory molecules on circulating B cells in GPA patients by employing spectral flow cytometry. We aimed to enhance our understanding of B-cell activation and tolerance breach in GPA and uncover insights that could lay the foundation for new therapeutic approaches and disease monitoring markers.

Using a 35-color B-cell-specific spectral flow cytometry panel, we gained an in-depth overview of the circulating B-cell compartment in GPA. GPA patients exhibited an aberrant B-cell subset distribution compared to HCs. For instance, the frequency of naive B cells within the total B-cell compartment was higher, while the frequency of memory B cells was lower in GPA patients compared to HCs. Although data on the B-cell subset distribution in GPA/AAV vary across studies, these findings are consistent with other studies and our previous observations [14, 34, 35]. Moreover, we identified several potentially interesting populations with our unsupervised analysis approach, including IgM and IgA-expressing memory B-cell populations. IgM^+^IgD^+^CD27^+^ memory B cells, representing marginal zone B cells [36], were found to be lower in active GPA compared to remission and HC, which is consistent with observations in RA [37] and SLE [38]. IgA^+^ switched resting memory, IgA^+^CD38^−/+^CD27^−/+^CD86^−/low^ switched activated memory, and IgA^+^ plasmablasts were also identified, with IgA^+^ plasmablasts being higher in active GPA than in remission and IgA^+^ switched resting memory higher in active GPA than in HC. Circulating PR3-ANCAs of both IgM [39, 40] and IgA [41, 42] isotypes have been reported in GPA/AAV, highlighting the potential role of IgM^+^ and IgA^+^ B cells in the disease. Furthermore, we observed a tendency towards a higher frequency of PR3-AF647^+^PR3-PE^+^ B cells, a population enriched for PR3-specific B cells, in GPA compared to HC, aligning with findings from other studies [13, 14]. We observed a median frequency of ~0.1% within the total circulating B-cell population of GPA patients which is lower than those reported previously in GPA (median: 1.11% [13] and 4.77% [14]). This discrepancy is likely due to the more specific PR3-BCR dual-staining technique employed in our study compared to the single-staining flow cytometry approaches used in previous studies [43]. The frequency is higher than what has been observed in RA (median: ~0.01%) [44]. This discrepancy could be attributed to variations in techniques, such as the dual-staining method for CCP2, where differentially labeled streptavidin and extravidin tetramers conjugated to biotinylated CCP2 were employed. Additionally, it could suggest potential differences in the frequency of autoantigen-specific B cells across autoimmune diseases.

**Figure 5:**
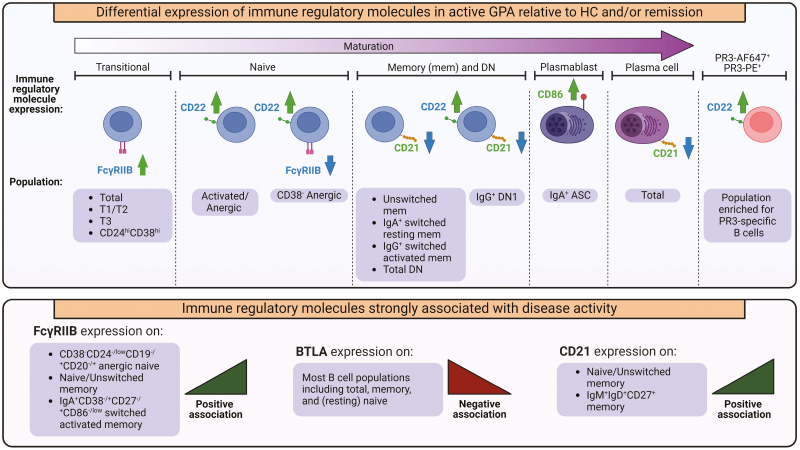
overview of the main findings in this study. Stimulatory immune molecules are depicted in green, and inhibitory immune molecules are depicted in blue. Green upward arrows indicate higher expression, while blue downward arrows indicate lower expression. DN: double negative; GPA: granulomatosis with polyangiitis; HC: healthy control; PR3: proteinase 3; ASC: antibody-secreting cells.

Our key findings include the differential expression of inhibitory and stimulatory immune molecules on B cells in GPA compared to HC, with the most notable differences observed in active GPA compared to both remission and/or HC (Fig. 5). Additionally, the expression of several regulatory immune molecules showed a strong association with disease activity.

The expression of FcγRIIB was upregulated on nonmature B cells, including total transitional, transitional T1/T2, transitional T3, and CD24^hi^CD38^hi^ B cells (a transitional population enriched for B regulatory cells [30]) in active GPA. Although other studies did not specifically focus on transitional B cells, altered FcγRIIB expression on B cells, primarily on antigen-experienced cells, appears to be prevalent in several autoimmune disorders. In a cohort of mostly MPA patients, lower FcγRIIB expression on circulating plasma cells was reported in both active and remission phases compared to HC, and the expression in active disease was inversely associated with disease activity and the percentage of cellular crescents in renal biopsies [26]. FcγRIIB expression is lower in active RA on circulating memory B cells and plasmablasts than HC and is associated with higher levels of autoantibodies [45]. In SLE, lower FcγRIIB expression on circulating CD27^+^ [22] and marginal zone B cells [23] is a characteristic of the disease. FcγRIIB is a receptor that inhibits BCR signaling upon binding the Fc region of IgG [46]. Studies on the expression and influence of FcγRIIB on the transitional B-cell stage in GPA and other autoimmune diseases are limited. Intriguingly, in an experimental mouse model of autoimmunity, increased expression of FcγRIIB on B cells decreased tolerance mechanisms at the immature and transitional B-cell stages, leading to reduced deletion and anergy of autoreactive B cells [47]. Conversely, overexpression of FcγRIIB in germinal center B cells strengthened tolerance mechanisms, while decreased expression reduced them. This suggests opposing effects of FcγRIIB on tolerance in nonmature versus antigen-experienced B cells. It has been hypothesized that overexpression of FcγRIIB may lower tolerance mechanisms by impacting the tonic signaling of immature/transitional B cells [47]. However, whether increased FcγRIIB expression on transitional B cells in GPA aids in breaching B-cell tolerance warrants further investigation. Together, these studies, along with our findings of altered FcγRIIB expression on B cells in GPA and the strong association of FcγRIIB expression on several B cells including CD38^−^CD24^−/low^CD19^−/+^CD20^−/+^ anergic naive, naive/unswitched memory, and IgA^+^CD38^−/+^CD27^−/+^CD86^−/low^ switched activated memory with disease activity, support a potential role for FcγRIIB in GPA pathogenesis.

Another remarkable observation in our study was the strong inverse association between the expression of the inhibitory immune molecule BTLA on most B-cell populations in active GPA and disease activity. In SLE, the expression of BTLA on naive B cells is inversely associated with SIGLEC-1 expression on monocytes, a type I IFN marker that contributes to the IFN signature which is related to disease activity in SLE, and autoantibody titers [24]. Interestingly, in the same study, the expression of BTLA on naive B cells was reduced compared to HC, and this dysregulation was functionally linked to a lack of inhibition of differentiation after BTLA engagement. Furthermore, B-cell tolerance is breached in mice lacking BTLA, which develop an autoimmune-like disease, including antibody production directed against nuclear antigens [48]. These studies imply that BTLA plays an important role in B-cell activation and maintaining B-cell tolerance. Further research on BTLA on B-cell function is needed to decipher the exact role of BTLA in GPA pathogenesis.

CD21 and CD86 expression were found to be altered on antigen-experienced B cells in active GPA. The expression of complement receptor 2/C3d receptor CD21 was reduced on IgG^+^ switched activated memory B cells, plasma cells, total DN B cells, and IgG^+^ DN1 B cells. Additionally, there was a strong positive association between CD21 expression on naive/unswitched memory B cells and IgM^+^IgD^+^CD27^+^ memory B cells with disease activity. A reduced expression of CD21 on circulating B cells is consistent with observations in several other autoimmune disorders and chronic inflammatory conditions, where an enrichment of CD21^−/low^ B cells has been reported (reviewed by Gjertsson *et al*. [49]). CD86 expression was found to be upregulated on IgA^+^ ASC plasmablasts indicating a higher activation status of these cells [50]. This finding further highlights the potential role of IgA^+^ B cells in the pathogenesis of GPA.

CD22 expression was elevated on PR3-AF647^+^PR3-PE^+^, CD38^-^ anergic naive, activated naive/anergic naive, and IgG^+^ DN1 B cells in active GPA. CD22 is an inhibitory receptor that binds α2,6-linked sialic acids which are present in eukaryotic cells including T cells, B cells, and monocytes [51]. In contrast to our findings, a reduction in CD22 expression on B cells has been mostly reported in several autoimmune diseases. For example, CD22 shows lower expression on various B-cell subsets, including transitional, naive, and particularly memory B cells in RA [52] and on total B cells in systemic sclerosis [53]. While the significance of this reduction is unknown, it could hypothetically lower the activation threshold since CD22 negatively regulates BCR signaling. The upregulation of CD22 we observed in GPA might be a regulatory feedback mechanism aimed at dampening the (auto)immune response during disease activity.

Finally, GPA patients in remission exhibited a distinct immune regulatory expression profile compared to HCs. Remission patients showed increased expression of CD21 on naive B cells and higher expression of FcγRIIB on a small naive/unswitched memory B-cell population. The factors leading to these alterations in immune regulatory molecule expression in remission are currently unknown. Potential contributors to these changes include the effects of prior treatments or ongoing low-level disease activity. Interestingly, since CD21 is a stimulatory molecule involved in influencing BCR signaling, the increased CD21 expression could potentially sensitize naive B cells for activation by complement. It is now well established that activation of the alternative pathway of the complement system is a key event in the pathogenesis of GPA/AAV [54]. Complement activation results in elevated circulating plasma levels and tissue deposition of various complement factors in GPA including C3d, the ligand of CD21 [55]. Moreover, multiple studies have demonstrated that ANCAs can induce neutrophil extracellular trap (NET) formation, a process in which neutrophils discharge nuclear chromatin [56]. NETs are extracellular complexes composed of chromatin coated with various proteins including the ANCA antigens PR3 and myeloperoxidase (MPO) but also complement components such as C3d [57]. Hence, these NETs might serve as an immunogenic scaffold that sustains the autoimmune response in AAV, potentially through C3d-CD21 sensitization of naive B cells. However, this hypothesis requires further investigation.

Several limitations of this study should be acknowledged. First, this was a single-center study in a relatively small cohort of GPA patients which may limit the generalizability of our findings. However, it is noteworthy that none of the patients were receiving immunosuppressive therapy at the time of blood collection. This unique aspect allowed us to assess the circulating immune profile while minimizing the confounding influence of such treatments, enhancing the relevance of our results despite the smaller sample size. Additionally, the B-cell distribution we observed in GPA is, in general, comparable to the current literature, suggesting that our cohort is representative. Second, the cross-sectional design of the study precluded the assessment of expression changes over time. In certain B-cell populations, the number of cells positive for the immune regulatory molecules investigated was low which restricted our ability to cover the whole B-cell compartment in GPA and HC. Furthermore, we focused on a specific set of immune regulatory molecules, potentially excluding other relevant molecules for B-cell activation. Moreover, while we report the detection of PR3-specific cells using a PR3-AF647/PR3-PE dual-staining approach, we did not formally confirm whether PR3-AF647^+^PR3-PE^+^ B cells are unequivocally PR3-ANCA-producing B cells.

In conclusion, our comprehensive analysis using spectral flow cytometry has provided novel insights into the expression patterns of key inhibitory and stimulatory immune molecules on B cells in GPA. The observed alterations in the expression of FcγRIIB, CD21, CD86, and CD22 on specific B-cell populations, along with the strong association of FcγRIIB, BTLA, and CD21 expression with disease activity, underscore the complexity of immune regulation in the pathogenesis of GPA. Future studies should focus on validating these findings in larger cohorts and exploring the underlying mechanisms driving the alteration of immune regulatory molecule expression in GPA and its consequences. This could lead to the identification of potential novel therapeutic targets and strategies. Additionally, longitudinal studies could provide insights into how the expression of immune regulatory molecules changes over the course of the disease and in response to therapy, thereby establishing their potential clinical utility.

## Supplementary data

Supplementary data are available at *Clinical and Experimental Immunology* online.

uxae096_suppl_Supplementary_Mateirals

uxae096_suppl_Supplementary_Files

## Data Availability

Spectral flow cytometry data from this study are accessible at http://flowrepository.org/id/FR-FCM-Z7PA. Other data are available upon request.

## Abbreviations:

AAV: ANCA-associated vasculitis
AC: active
ANCA: antineutrophil cytoplasmic antibodies
ASC: antibody-secreting cells
BAFFR: B-cell activating factor receptor
BCR: B-cell receptor
BH: Benjamini–Hochberg
BTLA: B and T lymphocyte attenuator
BVAS: Birmingham Vasculitis Activity Score
CRP: C-reactive protein
DN: double negative
eGFR: estimated glomerular filtration rate
FcγRIIB: Fc fragment of IgG receptor IIb
FDR: false discovery rate
GPA: granulomatosis with polyangiitis
HC: healthy control
ICOSL: inducible costimulator ligand
IL-6R: interleukin-6 receptor
IL-21R: interleukin-21 receptor
MFI: median fluorescence intensity
NET: neutrophil extracellular traps
OMIP: optimized multicolor immunofluorescence panel
PR3-ANCAs: proteinase 3-anti-neutrophil cytoplasmic antibodies
PBMCs: peripheral blood mononuclear cells
PB: plasmablasts
PC: plasma cells
PR3-BCR: PR3-binding B-cell receptor
RA: rheumatoid arthritis
RM: remission
SLE: systemic lupus erythematosus
TACI: transmembrane activator and CAML interactor
UMAP: Uniform Manifold Approximation and Projection
