# Supplementary material for: Circulating B cells display differential immune regulatory molecule expression in granulomatosis with polyangiitis
Source: Clin Exp Immunol. 2024 Oct 21;219(1):uxae096. doi: 10.1093/cei/uxae096 (PMC11773817; doi:10.1093/cei/uxae096)
Supplement: uxae096_suppl_Supplementary_Mateirals [file uxae096_suppl_Supplementary_Mateirals.pdf]

Supplemental data

Tables

Supplemental Table S1 | The 35-color B cell-specific spectral flow cytometry panel

Marker	Fluorophore	Clone	Supplier	Purpose
Viability	LIVE/DEAD Blue	-	Thermo Fisher Scientific	Dump gate
CD3	BV510	UCHT1	BioLegend	,,
CD14	BV510	MfP9	BD Biosciences	,,
CD19	cFluor BYG710	HIB19	Cytek	Lineage B cells
CD20	BV786	2H7	BD Biosciences	,,
CD38	PE/Fire 810	S17015F	BioLegend	,,
CD27	BV480	L128	BD Biosciences	,,
CD24	BUV395	ML5	BD Biosciences	,,
CD138	BUV496	MI15	BD Biosciences	,,
IgM	BV570	MHM-88	BioLegend	,,
IgD	BUV563	IA6-2	BD Biosciences	,,
IgG	BB515	G18-145	BD Biosciences	,,
IgA	VioBright FITC	IS11-8E10	Miltenyi Biotec	,,
CD21	APC-Vio770	HB5	Miltenyi Biotec	,,/Stimulatory molecule
CD10	APC-R700	HI10a	BD Biosciences	,,
CD43	BUV615	1G10	BD Biosciences	,,
CD86	BUV737	2331 (FUN-1)	BD Biosciences	,,/Stimulatory molecule
CD11c	BV650	3.9	BioLegend	,,
CXCR3	APC-Fire 810	G025H7	BioLegend	Migration
CXCR5	eFluor 450	MU5UBEE	Thermo Fisher Scientific	,,
BTLA	APC	MIH26	BioLegend	Inhibitory molecule
PD1	PE/Fire 640	EH12.2H7	BioLegend	,,
FcγRIIB	BV750	3D3	BD Biosciences	,,
CD22	BUV805	HIB22	BD Biosciences	,,
FcRL5	BB700	509F6	BD Biosciences	,,
CD5	BUV661	UCHT2	BD Biosciences	,,
CD40	PerCP-eFluor 710	5C3	Thermo Fisher Scientific	Stimulatory molecule
ICOSL	BB790-P	Custom	BD Biosciences	,,
BAFFR	BV605	11C1	BD Biosciences	,,
IL-21R	PE-Vio770	REA233	Miltenyi Biotec	,,
IL-6R	BV421	M5	BD Biosciences	,,
TACI	PE-Dazzle 594	1A1	BioLegend	,,
CD39	BV711	TU66	BD Biosciences	Regulatory B cells
PR3	AF647	-	-	PR3-specific B cells
PR3	PE	-	-	,,

Supplemental Table S2 | UMAP and FlowSOM settings

Phenotype of the manually gated B cells	$LD^{-}CD3^{-}CD14^{-}CD19^{+}CD20^{+/-}$
**UMAP**	
Markers included	CD24, CD138, IgD, CD43, CD5, CD86, CD22, CD185, CD27, IgM, CD11c, CD39, CD20, IgG, IgA, FcRL5, CD40, CD19, CD38, CD10, CD21, CD183
Subsampling (all B cells from all samples)	1.594.511
Number of neighbors	30
Minimum distance	0.4
Epochs	600
**FlowSOM**	
Number of clusters	35
Training iterations	10
Markers included	CD24, CD138, IgD, CD43, CD5, CD86, CD22, CD185, CD27, IgM, CD11c, CD39, CD20, IgG, IgA, FcRL5, CD40, CD19, CD38, CD10, CD21, CD183

Figures

A

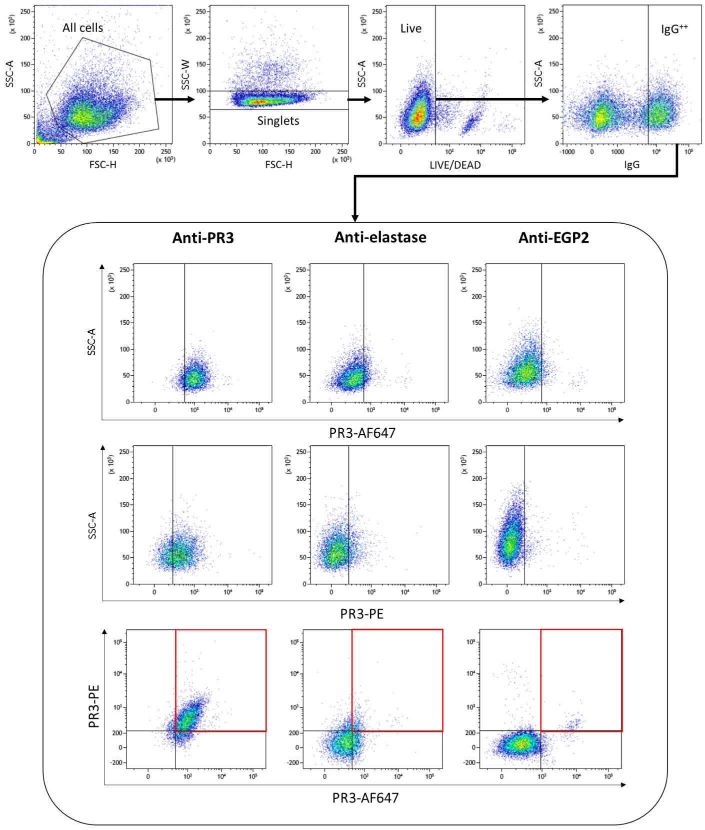

B

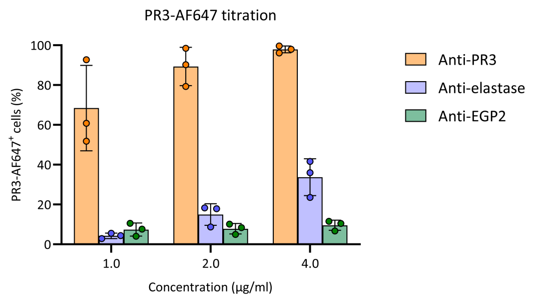

C

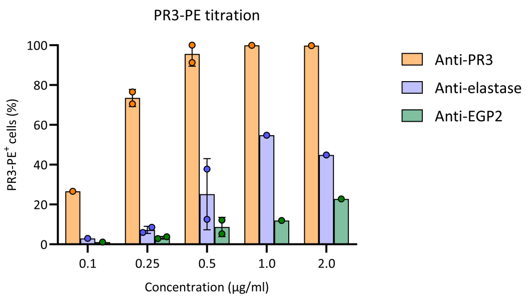

D

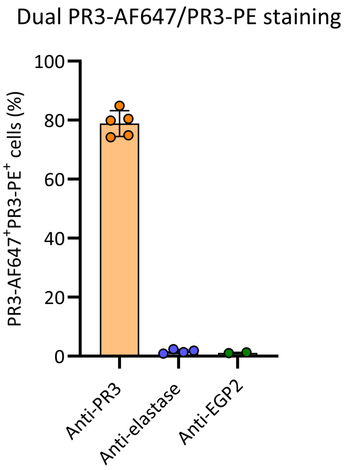

E

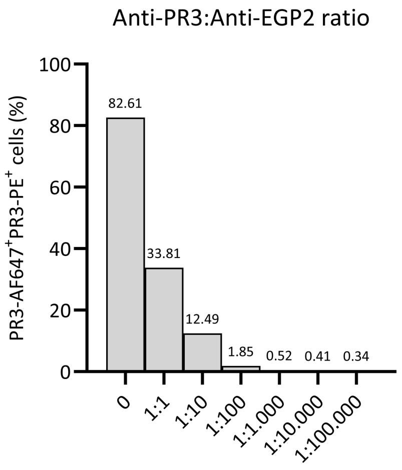

Supplemental Figure S1 | PR3-AF647 and PR3-PE specifically stain anti-PR3 hybridoma cells and combined reduce background staining.

Materials and methods

Fluorescent labeling of native human PR3: Native human PR3, originally isolated from leukocyte granules obtained from buffy coats of healthy individuals [1], was fluorescently labeled with AlexaFluor647 (AF647) or R-phycoerythrin (PE) using the AlexaFluor®647 Microscale Protein Labeling kit (Invitrogen, Grand Island, USA) and Lynx Rapid RPE Antibody Conjugation kit (Bio-Rad, Hercules, USA), respectively, according to the manufacturer's protocol. Native human PR3 was used instead of any other form of PR3 to maintain native (conformational) epitopes. AF647 and PE were chosen for the labeling of PR3 since they are considered the brightest fluorophores, which is necessary for the development of a high-sensitivity method for the detection of rare cell populations by flow cytometry [2].

Cell culture: Anti-human PR3, anti-human elastase (elastase belongs to the same family of serine proteases as PR3 [3]), and anti-human epithelial glycoprotein-2 (EGP2; non-PR3 and non-GPA related) hybridoma cells were maintained in RPMI 1640 supplemented with 10% FBS, 2.5% v/v Hybridokine (IQ products, Groningen, The Netherlands), 1% 100x ProHT (Lonza), 1 mM sodium pyruvate (Lonza), 60 μg/ml gentamicin, 2 mM L-glutamine (Lonza), and 0.05 mM β-mercaptoethanol (Merck, Darmstadt, Germany). 3T3-CD40L cells, a mouse fibroblast cell line characterized by surface expression and secretion of human CD40 ligand, were maintained in Dulbecco's Modified Eagle Medium/Nutrient Mixture F-12 (Gibco) supplemented with 10% FBS (Gibco), 2 mM L-Glutamine (Gibco), 10 mmol/l HEPES (Gibco), 15 μg/ml gentamicin, and 200 μg/ml G418 (Geneticin, Gibco).

Hybridoma staining: Hybridoma cells were washed twice with PBS after which 1.0×10^6 cells/ml were incubated with LIVE/DEAD™ Fixable Aqua Dead Cell dye (1:1.000, Invitrogen) for 30 min. at room temperature (RT). Cells were washed twice with PBS/1% bovine serum albumin (BSA) and 0.5×10^6 cells were subsequently stained with rat anti-mouse IgG1-BV605 (clone A58-1, BD Biosciences, San Jose, USA), PR3-AF647, or/and PR3-PE for 20 min., at RT. Afterward, cells were washed three times with PBS/1% BSA, and measurements were subsequently performed using a BD LSR II (BD Biosciences) or BD FACSymphony™ (BD Biosciences) flow cytometer. Unstained and single-stained samples were included as controls in each experiment. Data were analyzed using Kaluza Analysis Flow Cytometry Software (Beckman Coulter, Brea, USA) and GraphPad Prism version 10 (GraphPad Software, La Jolla, USA).

Results

(A) Representative flow cytometry plots of the gating of PR3-AF647$^+$, PR3-PE$^+$, and PR3-AF647$^+$PR3-PE$^+$ hybridoma cells (staining concentrations of 2.0 μg/ml PR3-AF647, 0.25 μg/ml PR3-PE, and 2.5 μg/ml PR3-AF647/0.25 μg/ml PR3-PE respectively). Gates were set on live IgG^{++} cells and defined using unstained samples. **(B)** Titration of PR3-AF647 on anti-human PR3, anti-human, and anti-EGP2 hybridoma cells (n=3). Percentages of PR3-AF647$^+$ cells are shown. **(C)** Titration of PR3-PE on anti-human PR3, anti-human elastase, and anti-human EGP2 hybridoma cells (n=1-2). Percentages of PR3-PE$^+$ cells are shown. **(D)** Dual staining of anti-human PR3, anti-human elastase, and anti-human EGP2 hybridoma cells with PR3-AF647 (2.5 μg/ml) and PR3-PE (0.25 μg/ml) (n=2-5). Percentages of PR3-AF647$^+$PR3-PE$^+$ cells are shown. **(E)** PR3-AF647/PR3-PE dual staining of anti-human PR3 hybridoma cells mixed with varying ratios of anti-human EGP2 hybridoma cells (n=1). Percentages of PR3-AF647$^+$PR3-PE$^+$ cells are shown.

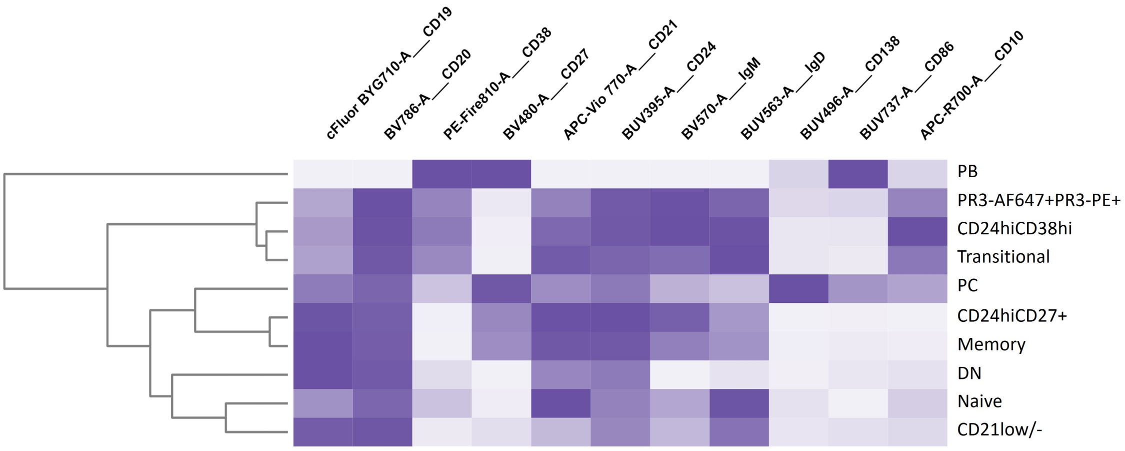

Supplemental Figure S2 | Heatmap visualizing marker expression profiles across manually gated B cell populations with min-max normalization for scaling, created using Ward's agglomerative hierarchical clustering.

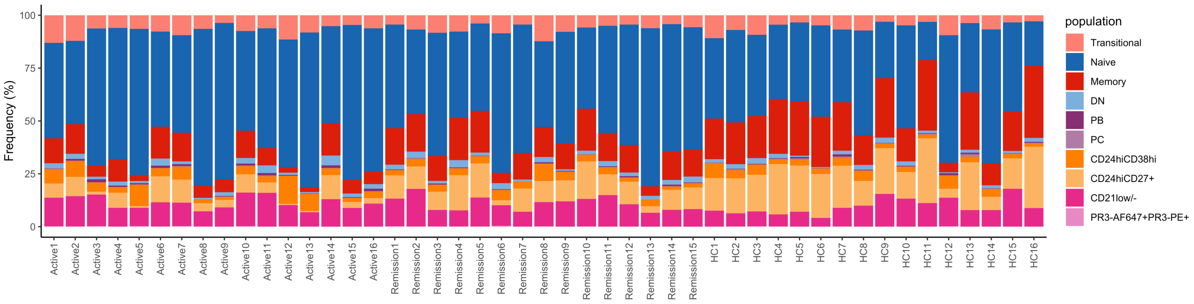

Supplemental Figure S3 | Frequency plot displaying the distribution of major B cell populations identified via manual gasting within the total B cell population across samples

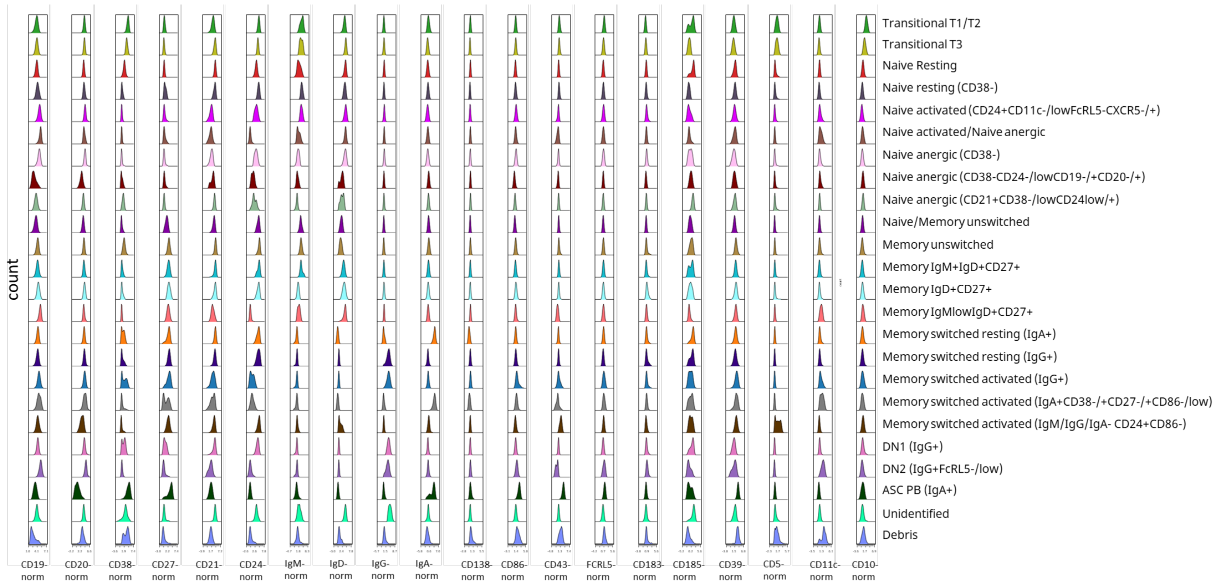

Supplemental Figure S4 | Histograms depicting the expression patterns of normalized markers used for UMAP cluster annotation

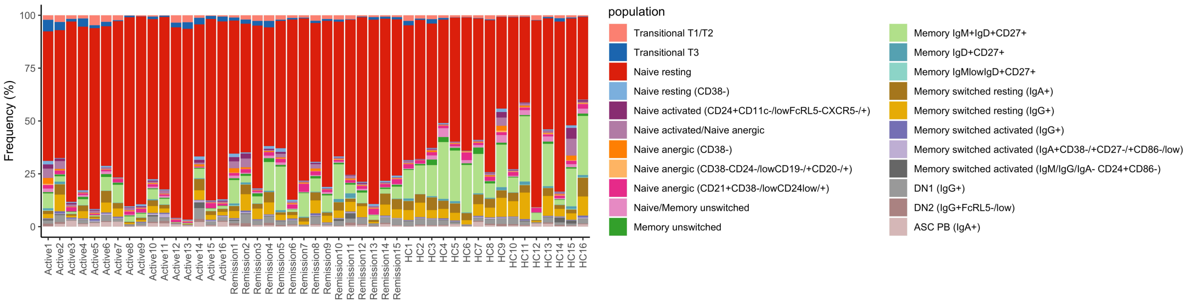

Supplemental Figure S5 | Frequency plot displaying the distribution of B cell populations identified via UMAP within the total B cell population across samples

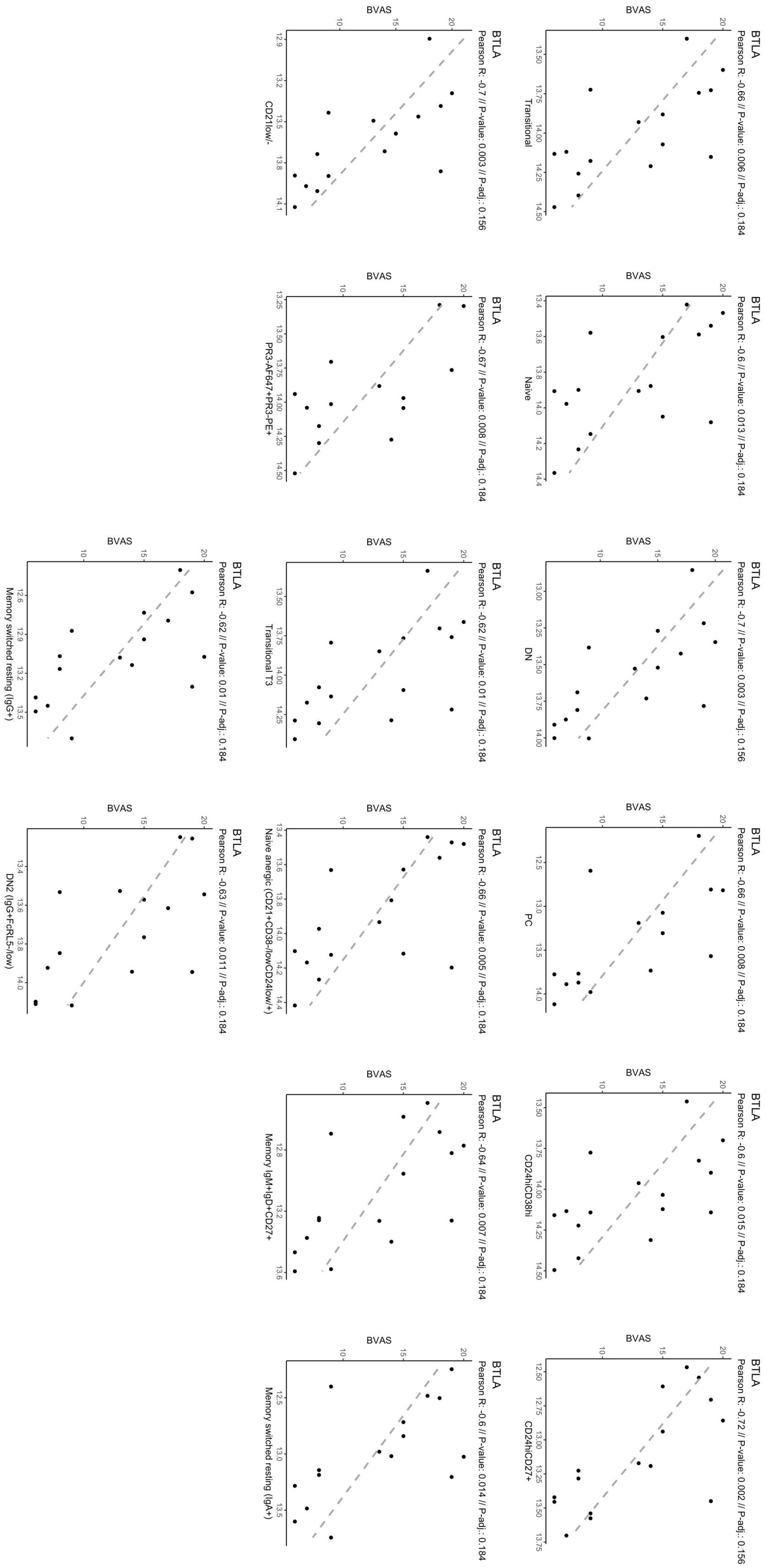

Supplemental Figure S6 | Scatter plots demonstrating the strong correlation of BTLA expression on various B cell populations with the BVAS

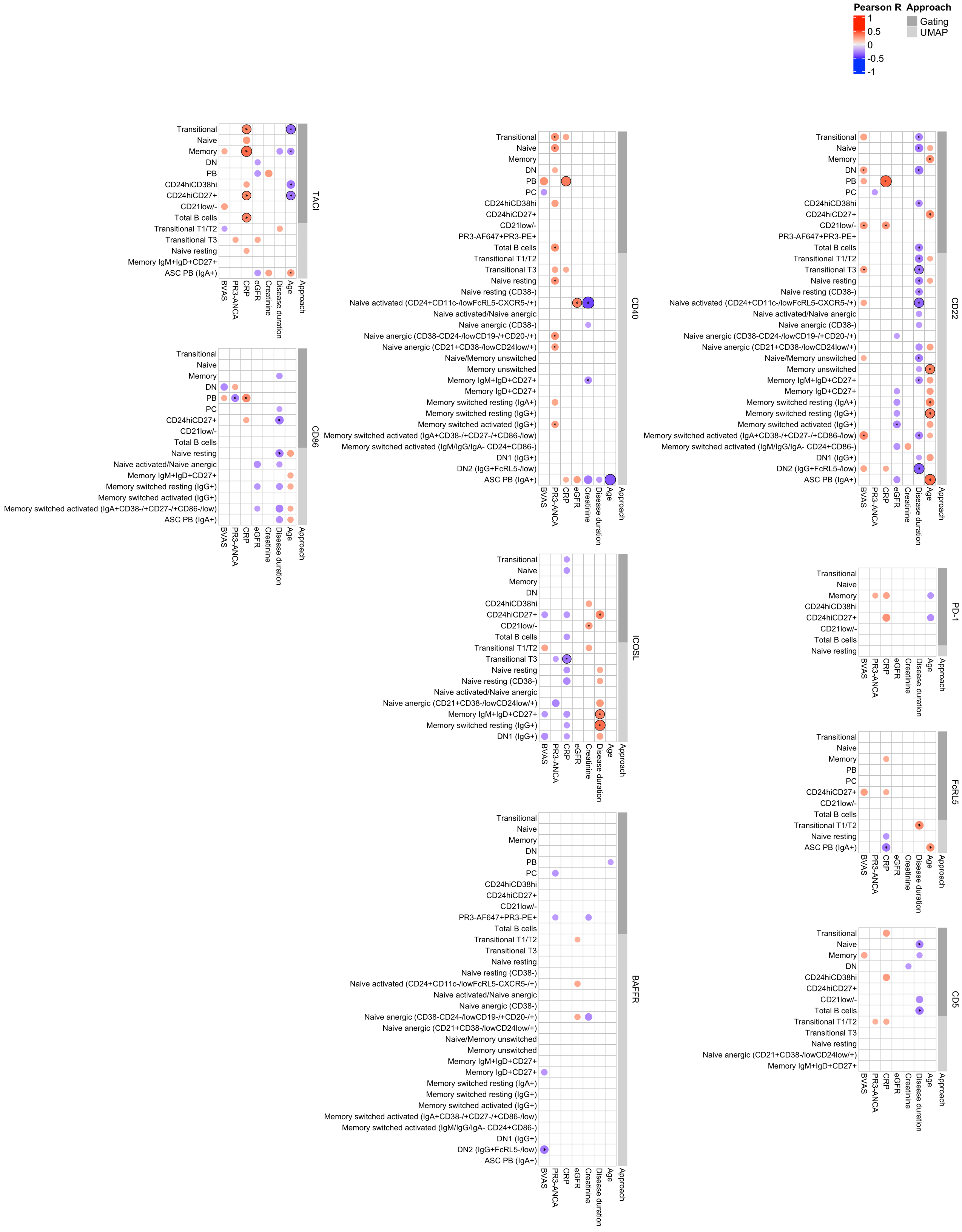

Supplemental Figure S7 | Correlation heatmaps displaying regulatory molecule expression on B cell populations in active GPA (n=16) with clinical parameters. The size of the dots is proportional to the correlation coefficient. Only correlations with coefficients ≥0.4 are shown, and those with coefficients ≥0.6 are highlighted with a black border. Asterisks indicate unadjusted *P*<0.05. For all *P*(adj)-values, see Supplemental data file 2.

References

[1] Y. M. Van Der Geld, W. Oost-Kort, P. C. Limburg, U. Specks, and C. G. M. Kallenberg, "Recombinant proteinase 3 produced in different expression systems: recognition by anti-PR3 antibodies," *J. Immunol. Methods*, vol. 244, no. 1–2, pp. 117–131, Oct. 2000, doi: 10.1016/S0022-1759(00)00261-1.

[2] J. Boonyaratanakornkit and J. J. Taylor, "Techniques to Study Antigen-Specific B Cell Responses," *Frontiers in immunology*, vol. 10. NLM (Medline), p. 1694, Jul. 24, 2019. doi: 10.3389/fimmu.2019.01694.

[3] B. Korkmaz, M. S. Horwitz, D. E. Jenne, and F. Gauthier, "Neutrophil Elastase, Proteinase 3, and Cathepsin G as Therapeutic Targets in Human Diseases," *Pharmacol. Rev.*, vol. 62, no. 4, p. 726, Dec. 2010, doi: 10.1124/PR.110.002733.
